# No evidence for *PALB2* methylation in high-grade serous ovarian cancer

**DOI:** 10.1186/1757-2215-6-26

**Published:** 2013-04-12

**Authors:** Thomas Mikeska, Kathryn Alsop, Gillian Mitchell, David DL Bowtell, Alexander Dobrovic

**Affiliations:** 1 Molecular Pathology Research and Development Laboratory, Department of Pathology, Peter MacCallum Cancer Centre, East Melbourne, VIC, Australia; 2 Department of Pathology, The University of Melbourne, Parkville, VIC, Australia; 3 Cancer Genomics and Genetics, Peter MacCallum Cancer Centre, East Melbourne, VIC, Australia; 4 Department of Biochemistry and Molecular Biology, The University of Melbourne, Parkville, VIC, Australia; 5 Familial Cancer Centre, Peter MacCallum Cancer Centre, East Melbourne, VIC, Australia; 6 Sir Peter MacCallum Department of Oncology, The University of Melbourne, Parkville, VIC, Australia

**Keywords:** DNA methylation, Ovarian cancer, Fanconi anaemia, *PALB2*, MS-HRM

## Abstract

**Background:**

High-grade serous ovarian cancers are a distinct histological subtype of ovarian cancer often characterised by a dysfunctional BRCA/Fanconi anaemia (BRCA/FA) pathway, which is critical to the homologous recombination DNA repair machinery. An impaired BRCA/FA pathway sensitises tumours to the treatment with DNA cross-linking agents and to PARP inhibitors. The vast majority of inactivating mutations in the BRCA/FA pathway are in the *BRCA1* and *BRCA2* genes and occur predominantly in high-grade serous cancer. Another member of the BRCA/FA pathway, *PALB2* (*FANCN*), was reported to have been inactivated by DNA methylation in some sporadic ovarian cancers. We therefore sought to investigate the role of *PALB2* methylation in high-grade serous ovarian cancers.

**Finding:**

*PALB2* methylation was investigated in 92 high-grade serous ovarian cancer samples using methylation-sensitive high-resolution melting analysis. DNA methylation of *PALB2* was not detected in any of the ovarian cancer samples investigated.

**Conclusion:**

Epigenetic silencing by DNA methylation of *PALB2* is not a common event in high-grade serous ovarian cancers.

## Findings

Ovarian cancer comprises several broad groups of distinct diseases [1]. The largest group are high-grade serous ovarian cancers, of which a substantial proportion are characterised by an impaired BRCA/Fanconi anaemia (BRCA/FA) pathway [2,3]. The BRCA/FA pathway is a key part of the homologous recombination DNA repair machinery and includes the *BRCA1* and *BRCA2* genes as well as members of the Fanconi anaemia complementation group. The inactivation of the BRCA/FA pathway is associated with an increased sensitivity of cancerous cells to DNA cross-linking agents and to PARP inhibitors [4,5].

The vast majority of inactivating mutations that occur in the BRCA/FA pathway in high-grade serous ovarian carcinomas are found in the *BRCA1* and *BRCA2* genes [2]. The protein product of the Fanconi anaemia gene *PALB2* (*FANCN*) serves as a bridge between BRCA1 and BRCA2 [6]. Mutations of *PALB2* have been associated with familial breast cancer and pancreatic cancer [6]. The occurrence of *PALB2* mutations in ovarian cancer has been less studied but is probably rare [7,8].

Aberrant DNA methylation is an alternative mechanism for *PALB2* inactivation. *PALB2* methylation has been reported in familial and sporadic breast cancer cases as well as in sporadic ovarian cancer samples [9]. In the sporadic ovarian cancer samples, *PALB2* methylation was reported to occur at a frequency of approximately 8%. However, the number of sporadic ovarian cancer cases investigated was quite small (53 samples) and consisted of different histological subtypes, grades and stages.

We sought to investigate aberrant *PALB2* methylation in a large number of high-grade serous ovarian cancers using methylation-sensitive high-resolution melting (MS-HRM) [10]. MS-HRM uses methylation-independent PCR primers which allow the amplification of bisulfite-modified templates independent of their methylation status. The analysis is based on the different melting behaviour of unmethylated and methylated templates after PCR amplification. The melting behaviour of an individual sample is visualised as a melting profile, which can be compared to melting profiles of DNA methylation standards and allows the estimation of the amount of methylation semi-quantitatively [11].

Ninety-two unselected high-grade serous ovarian cancer samples from The Australian Ovarian Cancer Study (AOCS) were used in this study. AOCS is a population-based case control study where newly diagnosed cases of ovarian, peritoneal and fallopian tube tumours were prospectively ascertained from major treatment centres and state-based cancer registries around Australia between January 2002 and June 2006, as previously described [3,12]. DNA was extracted from the fresh-frozen primary tumour samples using the DNeasy Blood and Tissue Kit (Qiagen, Hilden, Germany). Primary tissue sample assessed as being of low tumour content by pathological review was needle macro-dissected before DNA extraction. DNA concentration and quality was measured using the NanoDrop ND-1000 Spectrophotometer (NanoDrop Technologies, Thermo Fisher Scientific, Wilmington, DE). The use of the DNA has been approved by the Human Research Ethics Committee at the Peter MacCallum Cancer Centre. Fully methylated human control DNA was obtained commercially (Millipore, Billerica, MA). Control DNAs from the peripheral blood of normal individuals and the HL-60 cell line were extracted by using the QIAamp DNA Blood Mini Kit (Qiagen) according to the manufacturer’s instructions.

For bisulfite modification, 200 ng of DNA extracted from the high-grade serous ovarian cancer samples and 500 ng of the control DNAs were bisulfite modified using the EpiTect Bisulfite Kit (Qiagen) according to the manufacturer’s instructions. The bisulfite-modified DNA from the high-grade serous ovarian cancer samples was eluted twice in a final volume of 40 μL (50 μL for the control DNAs) of the supplied elution buffer, to give a theoretical concentration of 5 ng/μL (10 ng/μL for the control DNAs) presuming no loss of DNA during bisulfite modification.

*PALB2* and *RASSF1A* methylation was investigated by MS-HRM. DNA methylation standard series were prepared by diluting the bisulfite-modified fully methylated control DNA in bisulfite-modified unmethylated control DNA from peripheral blood for the analysis of *PALB2* methylation, and from HL-60 for the analysis of *RASSF1A* methylation, respectively. The amount of PCR amplifiable templates of the fully methylated and unmethylated control DNAs was normalised prior to dilution as previously described [13]. The DNA methylation standard series comprised 100%, 50%, 25%, 10%, and 0% of methylated control DNA. MS-HRM was performed on a Rotor-Gene 6000 (Corbett, Sydney, Australia). Each sample and each DNA methylation standard was run in duplicate, while the genomic DNA control and the no template control were run only once.

**Figure 1:**
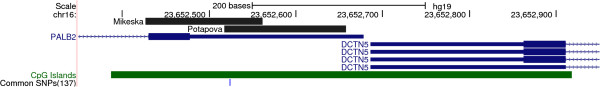
**Genomic positions of the PCR amplicons used in the Potapova study and in this study respectively, relative to exon 1 of *PALB2 *in the UCSC Genome Browser (GRCh37/hg19).** The region investigated in this study partially overlaps with the region analysed in the Potapova study [9].

The PCR primer sequences for the *PALB2* methylation analysis are: 5^′^-TTTTCGGTTTAGGGTTAATTGGGTT-3^′^ (forward primer) and 5^′^-CACCTTTTCCTTCTCCTCACAACTAAA-3^′^ (reverse primer). The PCR amplicon is 135 bp in size and corresponds to GenBank accession number AC008870.8, nucleotides 68,324 to 68,458 (UCSC Genome Browser: chr16: 23,652,428 to 23,652,562; GRCh37/hg19) (Figure 1). The amplicon contains 10 CpG dinucleotides and a G/A single-nucleotide polymorphism (rs8053188) between the primers.

PCR was performed in 0.1 mL tubes with a final reaction volume of 20 μL containing 200 nmol/L of the forward primer, 400 nmol/L of the reverse primer, 200 μmol/L of each dNTP, 5 μmol/L SYTO 9 (Life Technologies, Carlsbad, CA), 3.5 mmol/L MgCl_2_, 0.5U HotStarTaq DNA polymerase in its supplied buffer (1X) (Qiagen) and 5 ng (10 ng for the DNA methylation standard series) of bisulfite-modified DNA. PCR amplification was performed with one cycle of 95°C for 15 min, 50 cycles of 95°C for 20 s, 62°C for 20 s and 72°C for 30 s. This was immediately followed by a hold at 95°C for 1 min, 70°C for 1.5 min and a HRM step from 70 to 95°C rising at 0.2°C per second, and holding for 1 s after each stepwise increment. *RASSF1A* methylation analysis was performed as previously described [14] with an altered PCR amplification profile: one cycle of 95°C for 15 min, 55 cycles of 95°C for 10 s, 65°C for 20 s and 72°C for 30 s. This was immediately followed by a hold at 97°C for 1 min, 65°C for 1.5 min and a HRM step from 65 to 95°C rising at 0.2°C per second, and holding for 1 s after each stepwise increment.

**Figure 2:**
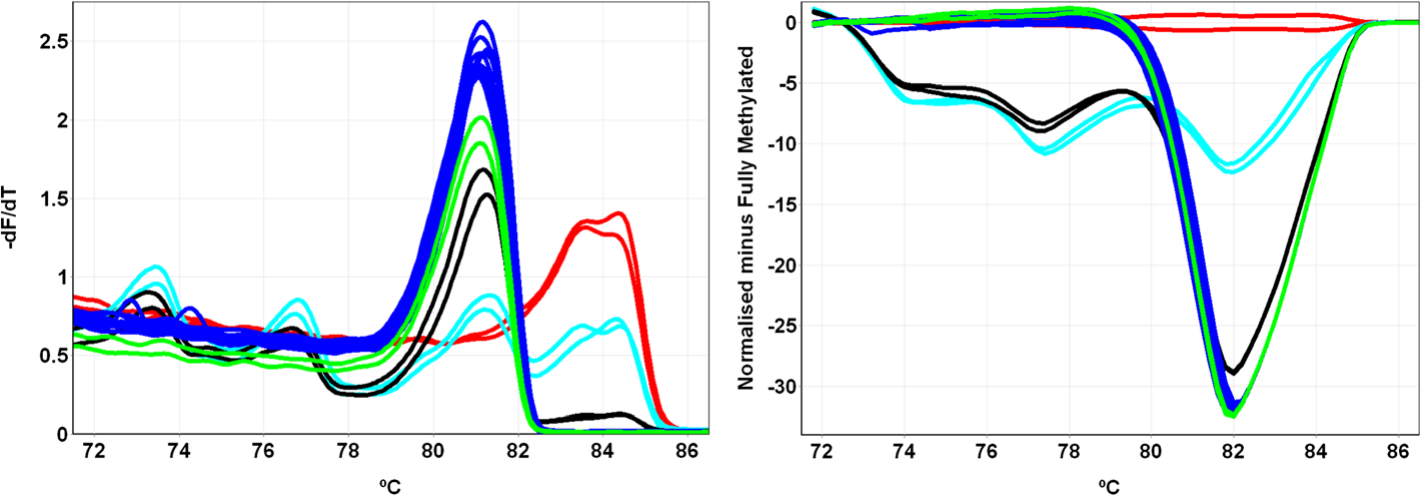
**Melting profiles of selected high-grade serous ovarian cancer samples obtained from MS-HRM experiments for *PALB2 *methylation analysis.** The melting profiles were partly smoothened by applying a light digital filter available in the Rotor-Gene 6000 analysis software. Melting profiles for fully methylated (100%), 50%, 10% and unmethylated (0%) standards as well as the tumour samples are shown as red, turquoise, black, green and blue curves. *T*m plots (negative first derivative of the melting curves) are shown in the left panel and the corresponding difference plots are shown in the right panel, respectively. In difference plots, the melting profiles of the fully methylated standard are chosen as a baseline and the relative differences in melting profiles of all other samples are plotted relative to this baseline. The high-grade serous ovarian cancer DNAs (blue curves) are unmethylated for *PALB2* as can be seen by their superimposition on the unmethylated standard (green curves).

The 92 high-grade serous ovarian cancer samples were investigated for aberrant *PALB2* methylation in exon 1 using MS-HRM. DNA methylation was not detectable in any of the high-grade serous ovarian cancer samples (Figure 2). To ensure that methylation would be detected if present, the samples were also tested for *RASSF1A* methylation which has been previously reported in high-grade serous ovarian cancer [15]. *RASSF1A* methylation was seen at various levels in a large proportion of the samples: 24 samples (26%) had no methylation, 46 samples (50%) had less than 10% methylation and 22 samples (24%) had methylation levels ranging from 10% to 90%.

Potapova *et al.* reported methylation of a region in exon 1 of *PALB2* in breast and ovarian cancers [9]. Quantitative methylation-specific PCR was used to detect aberrant *PALB2* methylation. DNA methylation of the methylation-positive samples was subsequently confirmed by direct bisulfite sequencing. The region investigated by MS-HRM partially overlaps with the above region and contains the two CpG dinucleotides of the reverse methylation-specific PCR (MSP) primer used in the previous study (Figure 1). A positive MSP result can not be obtained without one or both of those CpG dinucleotides being methylated. Thus our results have not been compromised by analysing an incompletely overlapping region to that previously analysed, which is a necessary consequence of the different PCR primer design principles for the two assays.

MS-HRM is a reliable and sensitive methodology which is well suited for the detection of homogeneous and heterogeneous methylation at gene-specific loci [16,17]. The *PALB2* MS-HRM assay conditions have been optimised and show sensitivity for the reliable detection of methylated epialleles down to below 10% (Figure 2). The sensitivity of the MS-HRM assay to detect aberrant DNA methylation is sufficient as it was estimated that some samples showed less than 20% of methylated epialleles [9].

Interestingly, all the four sporadic ovarian cancer cases that were found to be positive for *PALB2* methylation in the Potapova study were clear cell carcinomas, or showed foci of clear cell carcinoma [9]. Clear cell ovarian tumours have been compared to renal cell tumours in the past [18], and are now believed to be not only morphologically [19], but molecularly distinct when compared to high-grade serous and high-grade endometrioid ovarian cancers [20,21].

The lack of *PALB2* methylation-positive samples in our study might be explained by the fact that we have investigated high-grade serous ovarian cancer cases only, which presumably evolve via a different tumorigenic pathway of development than clear cell carcinomas [20]. However, because high-grade serous ovarian cancer is driven by disrupted homologous recombination, we may have reasonably expected to observe *PALB2* methylation as a method of pathway disruption.

In conclusion, we showed that epigenetic silencing by DNA methylation is an unlikely mechanism for *PALB2* inactivation in high-grade serous ovarian cancers. Our findings and those of The Cancer Genome Atlas Research Network [2] now challenge the contribution of *PALB2* methylation to the dysfunction of the BRCA/FA pathway in this histological subtype of ovarian cancer.

## Abbreviations

BRCA/FA: BRCA/Fanconi anaemia; HRM: High-resolution melting; MS-HRM: Methylation-sensitive high-resolution melting; PCR: Polymerase chain reaction.

## Competing interests

The authors declare that they have no competing interests.

## Authors’ contributions

TM developed the *PALB2* MS-HRM assay, performed the experiments, analysed and interpreted the data, and drafted the manuscript. KA performed the experiments, analysed the data, and contributed to the writing of the manuscript. DDLB and GM initiated the *Genotyping in the Australian Ovarian Cancer Study* project as well as the overarching project of which this investigation forms a part and contributed to the writing of the manuscript. AD supervised the work and co-wrote the manuscript. All authors read and approved the manuscript.

## Authors’ information

David DL Bowtell and Alexander Dobrovic are joint senior authors.
